# Expansion of cardiac ischemia/reperfusion injury after instillation of three forms of multi-walled carbon nanotubes

**DOI:** 10.1186/1743-8977-9-38

**Published:** 2012-10-16

**Authors:** Rakhee N Urankar, Robert M Lust, Erin Mann, Pranita Katwa, Xiaojia Wang, Ramakrishna Podila, Susana C Hilderbrand, Benjamin S Harrison, Pengyu Chen, Pu Chun Ke, Apparao M Rao, Jared M Brown, Christopher J Wingard

**Affiliations:** 1 Department of Physiology, Brody School of Medicine at East Carolina University, 600 Moye Blvd, Greenville, Brody 6N98, NC 27834, USA; 2 Department of Pharmacology and Toxicology, East Carolina University, Greenville, NC, 27834, USA; 3 Health Sciences, Institute for Regenerative Medicine, Wake Forest University, Winston- Salem, NC, 27157, USA; 4 Department of Physics and Astronomy, Clemson University, Clemson, SC, 29643, USA

**Keywords:** Pulmonary exposure, BAL, Serum cytokines, Modified carbon nanotubes, MWCNT, Nanotoxicology, Nanoparticle

## Abstract

**Background:**

The exceptional physical-chemical properties of carbon nanotubes have lead to their use in diverse commercial and biomedical applications. However, their utilization has raised concerns about human exposure that may predispose individuals to adverse health risks. The present study investigated the susceptibility to cardiac ischemic injury following a single exposure to various forms of multi-walled carbon nanotubes (MWCNTs). It was hypothesized that oropharyngeal aspiration of MWCNTs exacerbates myocardial ischemia and reperfusion injury (I/R injury).

**Methods:**

Oropharyngeal aspiration was performed on male C57BL/6J mice with a single amount of MWCNT (0.01 - 100 μg) suspended in 100 μL of a surfactant saline (SS) solution. Three forms of MWCNTs were used in this study: unmodified, commercial grade (C-grade), and functionalized forms that were modified either by acid treatment (carboxylated, COOH) or nitrogenation (N-doped) and a SS vehicle. The pulmonary inflammation, serum cytokine profile and cardiac ischemic/reperfusion (I/R) injury were assessed at 1, 7 and 28 days post-aspiration.

**Results:**

Pulmonary response to MWCNT oropharyngeal aspiration assessed by bronchoalveolar lavage fluid (BALF) revealed modest increases in protein and inflammatory cell recruitment. Lung histology showed modest tissue inflammation as compared to the SS group. Serum levels of eotaxin were significantly elevated in the carboxylated MWCNT aspirated mice 1 day post exposure. Oropharyngeal aspiration of all three forms of MWCNTs resulted in a time and/or dose-dependent exacerbation of myocardial infarction. The severity of myocardial injury varied with the form of MWCNTs used. The N-doped MWCNT produced the greatest expansion of the infarct at any time point and required a log concentration lower to establish a no effect level. The expansion of the I/R injury remained significantly elevated at 28 days following aspiration of the COOH and N-doped forms, but not the C-grade as compared to SS.

**Conclusion:**

Our results suggest that oropharyngeal aspiration of MWCNT promotes increased susceptibility of cardiac tissue to ischemia/reperfusion injury without a significant pulmonary inflammatory response. The cardiac injury effects were observed at low concentrations of MWCNTs and presence of MWCNTs may pose a significant risk to the cardiovascular system.

## Introduction

The last decade has witnessed tremendous advances in the field of nanotechnology, with the worldwide market for products produced using nanotechnology estimated to approach US $1 trillion by 2015 [1]. However, the growth in development of these materials is not matched by a parallel understanding of potential exposure and health risks [2].

Multi-walled carbon nanotubes (MWCNTs) are comprised of cylindrically arranged graphite sheets with a nanoscale diameter. They are manufactured predominantly by electrical arc discharge, laser ablation and chemical vapor deposition processes [3]. Carbon nanotubes have potential applications in medical and electronic devices, supercapacitors, batteries and in the automotive and aerospace industries [4]. Because of the rapid development and application of MWCNTs, numerous avenues of exposure to these materials exist, as does the potential for unforeseen biological impacts.

Myocardial ischemia (MI) is one of the largest killers of Americans. In 2006, mortality from MI in the United States was 141,462 [5]. Reperfusion of ischemic myocardium is an absolute necessity to salvage tissue from eventual death and for ultimate limitation of infarct size. However, reperfusion after ischemia is associated with pathologic changes that cause additional myocardial damage and may significantly contribute to myocardial infarction size. This so-called ‘reperfusion injury’ results from the activation of a series of inflammatory mediators [6].

Despite increasing use of carbon nanotubes there remains limited research on the safety and potentially detrimental effects to human health. In most existing studies of carbon nanotube exposure, MWCNTs have been shown to potentiate allergic, inflammatory and fibrotic pulmonary responses [7-9]. Consistent with other reports, we have recently demonstrated that mice instilled with MWCNTs exhibited increased inflammatory cell activation, collagen deposition and granuloma formation in the lung tissue which correlated with impaired pulmonary function [10,11].

Inspite of a rapidly increasing knowledge of the potential adverse pulmonary effects of carbon nanotubes, our knowledge of the impact of carbon nanotubes on the cardiovascular system lags behind and has largely been limited to studies of single-walled carbon nanotubes (SWCNTs). These reports provide evidence for microvascular endothelial cell dysfunction, acceleration of atherosclerotic plaque evolution and pulmonary and peripheral vascular thrombosis [12-14]. In addition, SWCNTs may alter the arterial baroreflex function thus affecting the regulation of autonomic cardiovascular control [15].

While the impact of exposure to engineered nanomaterials on the cardiovascular system is largely unknown, a growing body of evidence from epidemiological and toxicological studies provides a strong link between pulmonary exposure of ambient particulate matter and increased cardiovascular morbidity and mortality [16-18]. Therefore, based upon limited studies with SWCNTs and our understanding of the cardiovascular effects of ambient air pollution particles, and the role of inflammation in the cardiac response to ischemia and reperfusion, it is plausible that MWCNT exposure may exacerbate I/R injury in the myocardium. To date there is lack of definitive studies investigating the effect of pulmonary exposure to MWCNT and cardiac injury. Therefore, the present study was designed to evaluate the susceptibility to cardiac ischemia injury following exposure to MWCNTs. We report here, for the first time, a concentration- and time-dependent effect of an acute MWCNT exposure and the influence of the physico-chemical properties of the MWCNTs on cardiac ischemic reperfusion injury.

## Results

### Multi-Walled Carbon Nanotube Characterization

**Figure 1:**
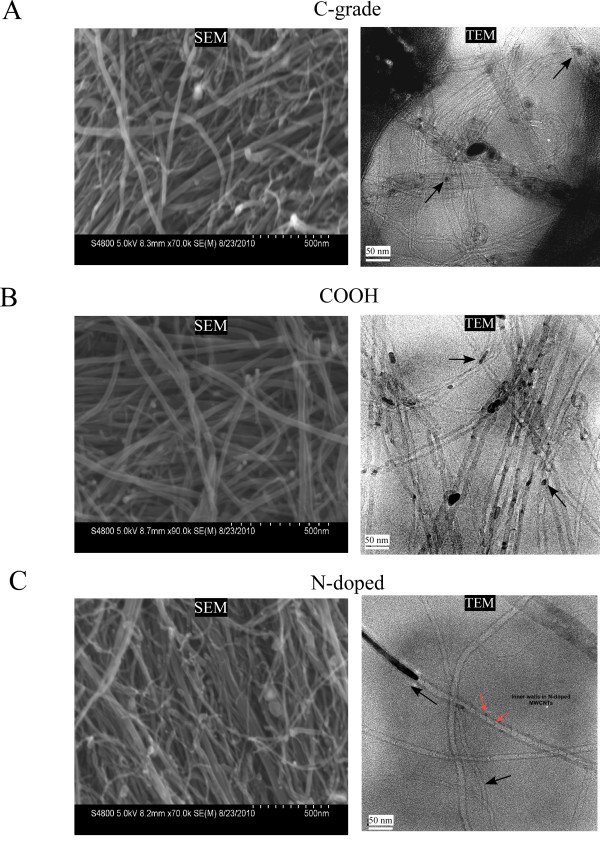
**Representative electron micrographs of the Commercial grade (C-grade, Panel A), carboxylated (COOH, Panel B) and Nitrogen doped (N-doped, Panel C) MWCNTs.** Left hand panels are scanning EM (SEM) of the bulk dry CNTs. Right hand panels are transmission EM (TEM) of MWCNT in suspension. Black arrows indicate presence of Fe metal catalyst inclusion within the MWCNT. Red arrows identify bamboo structure in N-doped MWNCTs.

All three forms of the MWCNTs used in this study had similar physical dimensions, with mean tube diameters ranging from 19 - 23 nm and overall length from 10 - 100 μm (Table 1 and Figure 1). Additional information detailing the diameter distributions and electron microscopy images of the bulk MWCNTs are provided in Additional file 1: Figures S2 - S10. Importantly, as shown in the transmission electron micrograph (Figure 1C), N-doped MWCNTs exhibit an internal structure different from pristine and COOH forms. TGA results for the 3 forms of bulk MWCNTs showed no initial mass loss, indicating that all samples were well dried before the analysis. The ash content for all 3 forms of MWCNT ranged between 3.7 - 5.1% weight (Table 1) with the ash appearance an orange-brown color, suggesting that their catalyst may be (Fe) iron-based. Subsequent x-ray adsorption spectral content analysis confirmed the present of Fe particles in the range of 0.04 - 0.07% atomic weight. Additionally, spectral analysis confirmed the functionalization by nitrogen doping (N-doped) and the higher oxygen content in the carboxylated (COOH) MWCNTs that were not present in the C-grade MWCNTs (Table 1 and Additional file 1: Figures S4, S7 and S10).

**Table 1:** MWCNT Characteristics

**MWCNT form**	**Mean Diameter (nm)**	**Length Range (μm)**	**Raman Band ratio (R)=I**_**D**_**/I**_**G**_	**Metal Ash Content by TGA (% weight)**	**Energy Dispersive spectra based concentrations**	**Surface area (m**^**2**^**/g)**	**Pore volume (cm**^**3**^**/g)**
C-grade	22.5 ± 1.3	10 - 100	0.29	4.80	C = 99.6 N = 0.0 O = 0.0 Fe = 0.04	113.10	0.69
COOH	19.1 ± 1.6	40 - 100	0.35	5.15	C = 96.5 N = 0.0 O = 3.5 Fe = 0.07	111.39	0.60
N-doped	23.3 ± 1.5	50 - 80	0.93	3.68	C = 80.8 N = 19.2 O = 0.0 Fe = 0.04	140.15	0.93

The presence of a strong disorder band (I_D_/I_G_ peak ratio) suggests the existence of structural defects as determined by Raman spectroscopy. Representative traces show the presence of the D and G band peaks and the ratios of the strengths of these bands (R = I_D_/I_G_) was used to quantify defects in the carbon nanostructure and reflect the defects in the functionalized COOH and N-doped versions of the MWCNTs (Additional file 1: FigureS1). The R values were relatively higher for carboxylated and N-doped samples confirming an increase in the intrinsic disorder due to acid functionalization and nitrogen-doping (Table 1).

The surface area of the three forms of MWCNTs were determined to be 113.10 m^2^/g for C-grade, 111.39 m^2^/g for COOH and 140.15 m^2^/g for the N-doped based on the BET equation [19]. The pore volume of the MWCNTs, defined as the ratio of the MWCNTs air volume to their total volume, was determined to be 0.69 cm^3^/g for C-grade, 0.60 cm^3^/g for COOH and 0.93 cm^3^/g for the N-doped utilizing the BJH method [20] (Table 1).

Two major peaks were observed in the hydrodynamic size distribution of each MWCNT suspension sample (Table 2). The C-grade MWCNTs displayed a major peak at 180 ± 50 nm, with a minor peak at 1100 ± 200 nm while both the COOH and the N-doped MWCNTs showed major peaks at 140 ± 50 nm, with a minor peak at 700 ± 150 nm. The slightly smaller mean size of the peaks for the COOH and N-doped MWCNT reflects the influence of the functionalization on the dispersibility of the MWCNTs. The larger sized peaks observed with the C-grade MWCNTs were likely due to bundling of the nanotubes through hydrophobic interaction and pi-stacking. Zeta potential and isoelectric point (IEP) are primary indicators for describing the surface charge and stability of a nanoparticle suspension. As reported in Table 2, at neutral pH, the zeta potentials of the C-grade, COOH and N-doped forms of the MWCNTs were in the range of - 37 to - 45 mV which suggested a good stability of these samples at neutral pH. The IEP of the MWCNT suspensions ranged from pH **+** 2.0 - 3.5 and thus at physiological pH the MWCNT suspensions were stable and not precipitating.

**Table 2:** MWCNT Suspension Characteristics

**MWCNT form**	**Zeta Potential**	**IEP**	**Hydrodynamic Size (Major and Minor Peak)**
C-grade	- 44.6 mV	3.5 pH	180 ± 50 nm and 1100 ± 200 nm
COOH	- 37.9 mV	2.0 pH	140 ± 50 nm and 700 ± 150 nm
N-doped	- 40.6 mV	3.0 pH	150 ± 50 nm and 1300 ± 250 nm

### Oropharyngeal Aspiration of MWCNT Induces a Modest Pulmonary Inflammatory Response

Pulmonary inflammation was assessed by determining BALF protein concentration and cell counts. We found elevated protein within the BALF with the 1 and 100 μg for all the three forms of MWCNTs at one day (1 day) post-oropharyngeal aspiration (Table 3). There was no difference in BALF protein levels between the SS and 3 forms of MWCNT at the later time points of seven day (7 day) and twenty-eight day (28 day) post-oropharyngeal aspiration (Table 3).

**Table 3:** Pulmonary Bronchoalveolar Lavage Fluid Protein (μg/ml)

**Treatment**	**Dose**	**1 Day Post Aspiration**	**7 Day Post Aspiration**	**28 Day Post Aspiration**
SS		0.07 ± 0.01	0.03 ± 0.01	0.15 ± 0.01
C-grade	1 μg	0.18 ± 0.01*	0.08 ± 0.01	0.12 ± 0.02
	100 μg	0.27 ± 0.04*†	0.12 ± 0.03	0.12 ± 0.01
COOH	1 μg	0.19 ± 0.02	0.07 ± 0.01	0.12 ± 0.01
	100 μg	0.30 ± 0.14	0.12 ± 0.03	0.12 ± 0.01
N-doped	1 μg	0.22 ± 0.02*	0.05 ± 0.01	0.17 ± 0.01
	100 μg	0.24 ± 0.04*	0.09 ± 0.01	0.19 ± 0.01

Bronchoalveolar lavage fluid (BALF) protein levels (μg/ml) following oropharyngeal aspiration of three forms of MWCNTs at 1μg and 100 μg or SS vehicle control (10% surfactant saline). The protein levels were performed on pooled aliquots of the BALF at 1 day, 7 day and 28 day post aspiration. Data reported as mean ± SEM for n = 4. * indicates significance from SS, † indicates significance from 1 μg dose within the same MWCNT form, *P* ≤ 0.05.

Modest pulmonary inflammation that consisted of increased numbers of macrophages and neutrophils was observed at 1 day and 7 day for all the three forms of MWCNTs (Table 4). In addition, eosinophils were present at 1 day following oropharyngeal aspiration of MWCNT, but waned by 7 days and were absent at 28 days. Eosinophil numbers were greatest with the oropharyngeal aspiration of the two functionalized forms of MWCNTs (*i.e*., COOH and N-doped). Overall, the BALF cell counts were generally higher in the 100 μg MWCNT than the 1 μg MWCNT.

**Table 4:** Pulmonary BALF Cell Counts at 1, 7 and 28 Days Following Oropharyngeal Aspiration of MWCNTs

**Treatment**	**Dose**	**Total Cells x 10**^**5**^	**Macrophages x10**^**5**^	**Neutrophils x 10**^**5**^	**Eosinophils x 10**^**5**^	**Lymphocytes x 10**^**5**^	**Epithelial Cells x 10**^**5**^
**1 Day**							
SS		1.80 ± 0.15	1.49 ± 0.12	0.01 ± 0.01	0.0 ± 0.0	0.0 ± 0.0	0.29 ± 0.03
C-grade	1 μg	2.25 ± 0.25	0.76 ± 0.08	1.65 ± 0.21	0.0 ± 0.0	0.0 ± 0.0	0.09 ± 0.01
	100 μg	4.64 ± 0.60*	1.12 ± 0.13	3.36 ± 0.71*	0.0 ± 0.0	0.0 ± 0.0	0.14 ± 0.05
COOH	1 μg	4.19 ± 0.45	1.42 ± 0.08	2.60 ± 0.38*	0.01 ± 0.01	0.0 ± 0.0	0.14 ± 0.02*
	100 μg	5.30 ± 1.57*	1.25 ± 0.35	3.92 ± 1.38*	0.08 ± 0.04	0.0 ± 0.0	0.03 ± 0.01*
N-doped	1 μg	7.16 ± 0.24*	2.06 ± 0.35	4.75± 0.27*	0.01 ± 0.01	0.0 ± 0.0	0.27 ± 0.04
	100 μg	6.22 ± 1.14*	1.84 ± 0.40	4.21 ± 0.75*	0.05 ± 0.02	0.0 ± 0.0	0.15 ± 0.01
**7 Day**							
SS		4.44 ± 0.15	4.20 ± 0.14	0.0 ± 0.0	0.0 ± 0.0	0.03 ± 0.01	0.19 ± 0.01
C-grade	1 μg	4.78 ± 1.21	4.46 ± 1.17	0.0 ± 0.0	0.0 ± 0.0	0.0 ± 0.0	0.31 ± 0.13
	100 μg	2.80 ± 0.72	2.53 ± 0.67	0.04 ± 0.01	0.03 ± 0.01	0.0 ± 0.0	0.18 ± 0.07
COOH	1 μg	1.33 ± 0.08	1.07 ± 0.08*	0.0 ± 0.0	0.0 ± 0.0	0.0 ± 0.0	0.25 ± 0.03
	100 μg	3.15 ± 0.05	2.63 ± 0.12*	0.19 ± 0.08	0.12 ± 0.06	0.03 ± 0.01	0.16 ± 0.01
N-doped	1 μg	5.61 ± 1.67	5.04 ± 1.52	0.0 ± 0.0	0.0 ± 0.0	0.06 ± 0.04	0.49 ± 0.16
	100 μg	4.92 ± 0.75	4.35 ± 0.86	0.03 ± 0.03	0.0 ± 0.0	0.07 ± 0.04	0.46 ± 0.09
**28 Day**							
SS		1.30 ± 0.24	1.24 ± 0.20	0.0 ± 0.0	0.0 ± 0.0	0.0 ± 0.0	0.06 ± 0.03
C-grade	1μg	1.04 ± 0.35	0.87 ± 0.26	0.0 ± 0.0	0.01 ± 0.01	0.0 ± 0.0	0.15 ± 0.07
	100 μg	1.48 ± 0.29	1.37 ± 0.27	0.0 ± 0.0	0.0 ± 0.0	0.01± 0.01	0.10 ± 0.02
COOH	1μg	1.29 ± 0.07	1.15 ± 0.08	0.0 ± 0.0	0.0 ± 0.0	0.0 ± 0.0	0.13± 0.02
	100 μg	1.74 ± 0.23	1.60 ± 0.22	0.02 ± 0.01	0.0 ± 0.0	0.0 ± 0.0	0.10 ± 0.01
N-doped	1 μg	0.92 ± 0.29	0.86 ± 0.28	0.0 ± 0.0	0.0 ± 0.0	0.06 ± 0.04	0.05 ± 0.01
	100 μg	1.27 ± 0.11	1.15 ± 0.10	0.0 ± 0.0	0.0 ± 0.0	0.07 ± 0.04	0.11 ± 0.01

Bronchoalveolar lavage fluid cell counts following oropharyngeal aspiration of three forms of MWCNTs at 1μg and 100 μg or SS vehicle control (10% surfactant saline). The cell counts were performed at 1 day, 7 day and 28 day post-instillation. Data reported as mean ± SEM for n = 4. * indicates significance from SS, † indicates significance from 1 μg dose in same MWCNT form, *P* ≤ 0.05.

**Figure 2:**
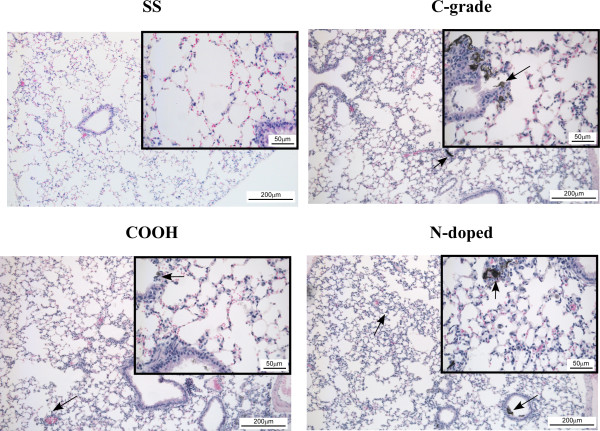
**Representative histological sections of the left lung 1 day post oropharyngeal aspiration of vehicle control (SS) and 100 μg of each of the MWCNT forms: Commercial grade (C-grade), Carboxylated (COOH) and Nitrogen doped (N-doped).** Images are of H&E stained 8μm thick cross-sections of left lung. Larger panel images were captured at 10x magnification and inset images at 40x magnification. Arrows indicate deposits of MWCNT.

Lung histopathology revealed modest alveolar and peribronchial inflammation with cellular infiltration of the alveolar septae at 1 day post-oropharyngeal aspiration of 100 μg MWCNTs (Figure 2). The presence of MWCNT agglomerates at various locations in the lung was observed for all three forms of MWCNTs. The absence of any striking differences in agglomeration trends of various MWCNTs was expected since all the MWCNT samples exhibited similar zeta potential values. Similar histological and inflammatory responses were seen at 1 μg MWCNTs at 1 day post oropharyngeal aspiration (Additional file 1: Figures S11 - S13). There appears to be a waning of the general inflammatory response in the lungs at 7 days following oropharyngeal aspiration of MWCNT. However, at 7 and 28 days post-oropharyngeal aspiration the development of granuloma-like lesions around deposits of MWCNTs appeared with 100 μg (Additional file 1: Figures S12 andS13).

### Effect of Oropharyngeal Aspiration of MWCNT on circulating leukocytes, red blood cells and platelets

Total and differential leukocyte counts were performed after oropharyngeal aspiration of 100 μg of each of the three forms of MWCNTs 1 day post-oropharyngeal aspiration and showed increased number of total leukocytes (Table 5). The Neutrophil count in the COOH group was statistically significant larger than the SS, the C-grade and the N-doped MWCNTs groups. We did not observe any statistically significant differences in the total red blood cell or the platelet counts between the SS and the three forms MWCNTs (Table 5).

**Table 5:** Blood Cell Counts 1 Day post Oropharyngeal Aspiration of 100 μg MWCNTs

**WBC Type/Treatment**	**SS**	**C-grade**	**COOH**	**N-doped**
Total WBC Count (10^3/μl)	4.18 ± 0.27	3.75 ± 0.45	5.03 ± 0.68	4.4 ± 0.21
Neutrophils	0.45 ± 0.06	0.40 ± 0.08	0.81 ± 0.08 *†#	0.40 ± 0.03
Lymphocytes	3.49 ± 0.24	3.04 ± 0.33	3.78 ± 0.56	3.72 ± 0.17
Monocytes	0.19 ± 0.03	0.22 ± 0.05	1.13 ± 0.86	0.25 ± 0.05
Eosinophils OO	0.03 ± 0.01	0.07 ± 0.01	0.04 ± 0.01	0.01 ± 0.01
RBC Count (10^6/μl)	8.49 ± 0.22	8.82 ± 0.28	8.91 ± 0.23	8.74 ± 3.07
PTL Count (10^3/μl)	1091.5 ± 79.8	771.3 ± 151.1	977.3 ± 102.3	980.3 ± 66.0

Blood Cell Counts following oropharyngeal aspiration of three forms of MWCNTs at 100 μg or SS vehicle control (10% surfactant saline). The cell counts were performed at 1 day post aspiration. Data reported as mean ± SEM for n = 6. * indicates significance from SS, † indicates significance from C-grade and # from N-doped MWCNT by one way ANOVA, *P* ≤ 0.05.

### Effect of Oropahryngeal Aspiration of MWCNT on Circulating Cytokine Levels

Because we were interested in determining if any circulating inflammatory mediators might be associated with the susceptibility of the cardiac tissue to ischemic damage, we evaluated serum levels of selected cytokines that previously have been described in pulmonary responses to particulate and MWCNT exposure, or cardiac ischemia and reperfusion [7,21]. Serum cytokine analysis was performed on blood samples collected at 1 day post-oropharyngeal aspiration of 100 μg MWCNTs. We found no significant difference in serum cytokine between the SS exposed mice and the C-grade MWCNT exposed mice (Table 6). There was a significant increase in serum levels of eotaxin following pulmonary exposure to COOH MWCNTs with increased levels of IL-6, IL-10, IL-12(p70) and KC (keratinocyte derived chemokine) following oropharyngeal aspiration of COOH MWCNT when compared to SS (Table 6). While oropharyngeal aspiration of the N-doped MWCNTs did not lead to any significantly increased circulating levels of these cytokines, there was small non significant elevations of IL-10, IL-12(p40), IL-13 and MIP1α (Table 6). Additional ELISA based analysis of eotaxin, Il-6, IL-1β and TNF- α were conducted in a separate set of rat serum from each experimental group (n = 6) as verification of of multiplex results. The re-evaluation for eoxtaxin and IL-6 were not different from the original values and were not reported. The result from the IL-1β assay revealed similar variability in the cytokine levels within any group as the multiplex data without any statistically significant differences between the groups while TNF-α was undetectable (Table 6).

**Table 6:** Serum Cytokine Profile 1 Day post Oropharyngeal Aspiration of 100 μg MWCNTs (pg/ml)

**Cytokine/Treatment**	**SS**	**C-grade**	**COOH**	**N-doped**
Eotaxin	306.4 ± 75.0	276.9 ± 42.3	903.8 ± 278.6*	302.6 ± 22
IL-5	16.3 ± 1.1	30.3 ± 10.0	11.6 ± 3.0	51.2 ± 28.3
IL-6	9.2 ± 1.3	16.1 ± 7.8	137.8 ± 66.0	42.4 ± 26.4
IL-10	8.5 ± 1.9	8.6 ± 3.4	24.6 ± 15.4	125.8 ± 118.1
IL-12 (p40)	17.5 ± 3.6	20.3 ± 5.3	16.3 ± 2.2	12.6 ± 2.2
IL-12 (p70)	35 ± 5.9	55.3 ± 34.9	22.9 ± 3.1	516.7 ± 464.5
IL-13	446.3 ± 41.6	326.9 ± 38.2	365.3 ± 45.6	848.3 ± 490.0
KC	173.2 ± 32.6	97.1 ± 7.1	645.1 ± 441.3	183.5 ± 53.1
MCP-1	39.6 ± 12.3	36.6 ± 8.6	46.6 ± 6.0	136.1 ± 104.4
MIP1α	38.2 ± 9.3	71.1 ± 4.5	42.0 ± 4.4	128.1 ± 86.9
MIP-1β	129.0 ± 11.4	70.2 ± 22.3	82.8 ± 29.6	136.2 ± 48.8
MIP-2	155.4 ± 27.7	86.4 ± 43.8	209.1 ± 38.9	102.4 ± 26.4
RANTES	30.7 ± 5.8	36.1 ± 6.4	57.5 ± 12.0	65.9 ± 38.5
†IL-1β	33.25 ± 3.83	222.86 ±114.71	127.66 ± 45.32	47.25 ± 7.36
†TNF-α	N.D.	N.D.	N.D.	N.D.

Serum cytokine profile by Lincoplex Cytokine/Chemokine 20 plex kit 1 day post oropharyngeal aspiration of three forms of MWCNTs at 100 μg and vehicle control SS (10% surfactant saline). Data reported as mean ± SEM for n = 4. * indicates significance from SS, *P* ≤ 0.05.

† IL-1β and TNF-α profile 1 day post oropharyngeal aspiration of three forms of MWCNTs at 100 μg and vehicle control SS (10% surfactant saline) by ELISA. Data reported as mean ± SEM for n = 6. N.D. not detected, *P* ≤ 0.05 by one way- ANOVA.

### MWCNTs Exacerbate Myocardial I/R Injury at 1, 7 and 28 Days Following Oropharyngeal Aspiration

**Figure 3:**
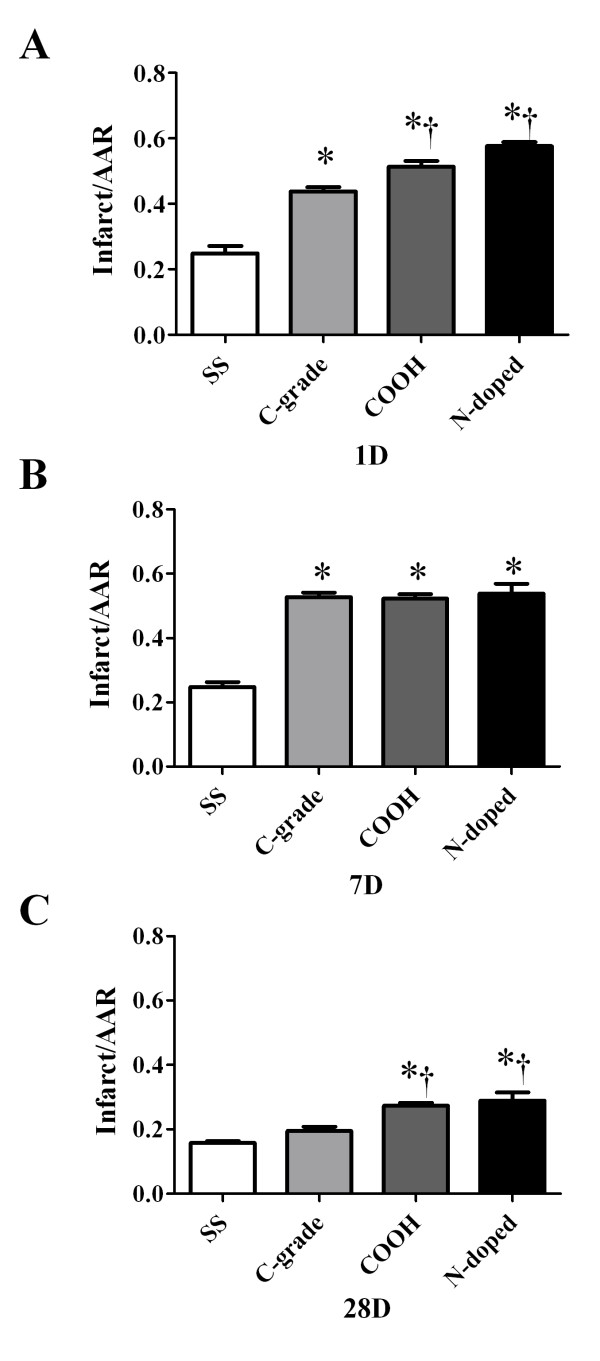
**The time-dependent cardiac ischemic-reperfusion (I/R) injury following vehicle control 10% surfactant/saline (SS) and 100 μg of each of the three forms of MWCNT: Commercial grade (C-grade), carboxylated (COOH) and nitrogen doped (N-doped).** The cardiac I/R injuries were performed at 1 day (**A**), 7 day (**B**) and 28 day (**C**) post oropharyngeal aspiration of 100 μl of the MWCNT suspension. Data are reported as mean ± sem with an n = 6. * indicates statistical significance from the SS group and † represent statistical significance from the c-grade group by ANOVA with TUKEY post hoc analysis with significance at p < 0.05.

To determine whether there was a time-dependent effect on myocardial I/R injury following oropharyngeal aspiration of MWCNTs, this exposure apporach was performed in mice with 100 μg C-grade, COOH or N-doped MWCNTs suspended in 100 μl of SS. The mice were then subjected to the cardiac ischemic reperfusion (I/R) injury protocol at 1 day, 7 days and 28 days post-oropharyngeal aspiration. At 1 day following oropharyngeal aspiration of 100 μg each of three forms of MWCNTs, coronary artery occlusion resulted in a ≥ 2-fold increase in left ventricle infarct size (Figure 3A). The oropharyngeal aspiration of the COOH and N-doped MWCNTs resulted in a significantly greater infarct size than the C-grade MWCNTs (Figure 3A). The expansion of the infarct remained present at 7 days following oropharyngeal aspiration of each of the three forms of MWCNTs (Figure 3B). By 28 days post-oropharyngeal aspiration, a decrease in infarct expansion was observed in all three groups, (Figure 3C). Overall, the N-doped and COOH MWCNT exposed mice displayed an early and sustained elevation in extent of I/R injury that was reduced but still evident and significantly elevated at 28 days, while the C-grade MWCNT exposed mice showed an elevated infarct size at 1 and 7 days following oropharyngeal aspiration, but not significantly elevated 28 days following oropharyngeal aspiration of MWCNT.

### Concentration-Dependent Exacerbation of Myocardial I/R Injury Following Oropharyngeal Aspiration of MWCNTs

**Figure 4:**
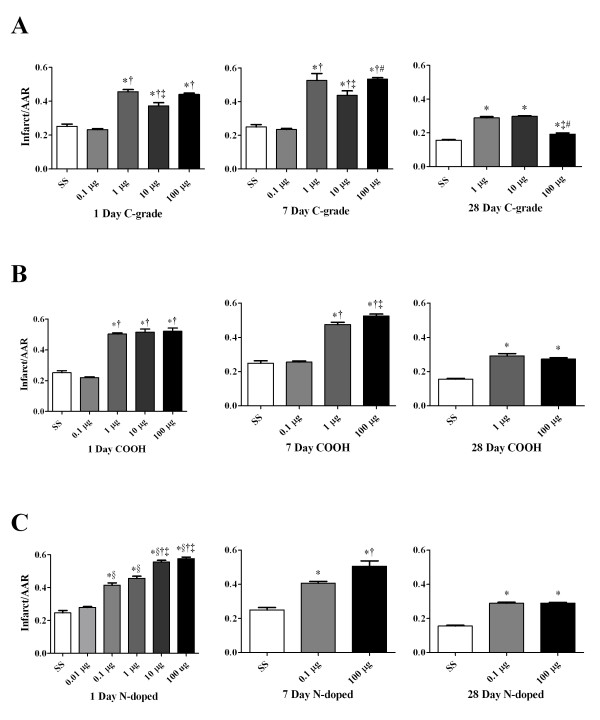
**Graphs depicting the extent time- and dose-dependent cardiac ischemic reperfusion (I/R) injury following oropharyngeal aspiration of vehicle control 10% surfactant/saline (SS) and 3 forms of the MWCNT: Commercial grade (C-grade) Row A, carboxylated (COOH) Row B and nitrogen doped (N-doped) Row C.** The cardiac I/R injuries were performed at 1 day (left most panels), 7 day (middle panels) and 28 day (right most panels) post aspiration of 100 μl of the MWCNT suspension. Data are reported as mean ± sem with an n = 6. * indicates statistical significance from the SS group, Â§ from 0.01 μg, † from 0.1 μg ‡ from 1.0 μg and # from 10 μg dosing as determined by ANOVA with TUKEY post hoc analysis with significance at p < 0.05.

Since oropharyngeal aspiration of 100 μg MWCNT resulted in significant infarct expansion, we attempted to determine if there was a concentration-dependent cardiac I/R injury response to MWCNTs. Oropharyngeal aspiration was performed on mice with each form of MWCNTs between 0.01 μg and 100 μg and I/R injury was assessed at 1, 7 and 28 days post-oropharyngeal aspiration (Figure 4). At 1 day post-oropharyngeal aspiration of C-grade and COOH MWCNTs, there was a significant exacerbation in the infarct size at 1 μg and larger as compared to the SS group (Figure 4A and B). For the C-grade and COOH MWCNT exposed mice, a no observable effect level was found at 0.1 μg MWCNTs (Figure 4A and B). Since the C- grade and the COOH MWCNTs displayed no statistical significant differences from S/S for 0.1 μg at 1 day and 7 days we chose not to examine the 0.1 μg at 28 days. However, at one day following oropharyngeal aspiration, the N-doped MWCNT exposed mice had a significantly elevated infarct size at 0.1 μg and a no observable effect level was found at 0.01 μg MWCNT (Figure 4C). Because there significant elevations in I/R injury area with 0.1 μg the 1 and 10 μg concentrations were not examined in the 7 and 28 day time points. Oropharyngeal aspiration of the C-grade and COOH forms of MWCNTs did not demonstrate a concentration-response relationship but rather exhibited a threshold effect of infarct expansion; only with the oropharyngeal aspiration of N-doped MWCNT did there appear to be a concentration -response relationship of mass dose and infarct size.

Based on the results of the 1 day MWCNT oropharyngeal aspiration experiments, we also examined a limited concentration-response series at 7 days and 28 days for all forms of the MWCNTs. The C-grade MWCNT I/R injury response showed persistent expansion of the infarct at concentrations of 1 and 10 μg at 7 and 28 days while the 100 μg concentration remained elevated at 7 days, but waned and was no longer statistically different from SS instilled mice at 28 days post- oropharyngeal aspiration (Figure 4A). The COOH MWCNT exposed mice maintained a significant expansion of the infarct size at 1 and 100 μg at both 7 and 28 days post- oropharyngeal aspiration. However, there was also a waning of the magnitude of the infarct expansion between 7 days and 28 days (Figure 4B). The I/R injury response with the N-doped MWCNT exposed mice also displayed a similar pattern with the lowest concentration (0.1 μg) that was significant at 1 day and remained persistent at both 7 days and 28 days but was smaller in magnitude at the later time point (Figure 4C).

## Discussion

Due to the diverse commercial utility of MWCNTs and the limited knowledge about their potential health risks on the cardiovascular system, the effect of pulmonary MWCNT exposure on an imposed cardiac stress was evaluated. We report, for the first time, that oropharyngeal aspiration of three different forms of MWCNTs promotes an increased susceptibility of cardiac tissue to ischemic reperfusion (I/R) injury. These effects were observed at a concentration as low as 0.1 μg MWCNT per animal and persisted for 28 days following a single oropharyngeal aspiration. We suggest that a pulmonary exposure to MWCNTs may pose a significant risk to the injured heart, particularly at low doses where overt pulmonary toxicity is not observed.

The safety of MWCNTs is still in debate due to the lack of systematic and complete toxicity evaluation as well as a limited understanding of the human exposure levels in occupational settings [22]. Current proposed guidelines by the National Institute for Occupational Safety and Health (NIOSH) limits exposure to 7 μg/m^3^, which is the lowest detectable level of airborne CNT by the latest analytical method [23]. The present study involved delivering a single defined amount of MWCNTs in a log order by oropharyngeal aspiration and examining pulmonary and cardiovascular endpoints at multiple time points. The utilization of such acute high concentrations to probe potential effects is a standard approach in toxicology and experimental pathology for initially surveying adverse effects [24]. While oropharyngeal aspiration provides a natural route of entry into the host and has been a method for the introduction of a variety of toxicants into the lungs [25] there is continued discussion regarding the real world relevance of this route of exposure. The pulmonary toxicity effects of CNT aspiration have shown to be comparable between inhalation and oropharyngeal aspiration [26]. Even with these caveats the chosen oropharyngeal aspiration approach allows primary responses to substance exposure to be studied independent of the compensatory mechanisms that may come into play with chronic exposures.

Evidence accumulated from epidemiological studies confirms a significant pathogenic correlation between the inhalation of small and ultrafine sized particles, including nano-sized particles from ambient air and cardiovascular events, such as angina, arrhythmia, ischemic heart failure and sudden death [27]. Epidemiologic and experimental studies have suggested an association between respiratory exposure to ambient ultrafine particles (including particles with a diameter < 100 nm) and the progression of cardiovascular disease [17,28]. A number of studies have also shown that pulmonary inhalation of ultrafine particulate matter generated from synthetic nanoparticles resulted in development of vascular abnormalities, including atherosclerosis, increased vascular tone and impaired endothelial-dependent vascular dilation [14,29-31]. While epidemiological and inhalation studies have provided indirect evidence that ambient particle inhalation exposure may be a risk factor for adverse cardiac events [12,27,32], no definitive study has identified a link between exposure to MWCNTs and a negative impact on the myocardium.

Various toxicological studies have demonstrated that pulmonary deposition of MWCNTs caused an acute and chronic pulmonary toxicity in animal models [7-9,11]. Pulmonary responses to MWCNT exposure include an acute inflammatory phase, a progressive fibrotic response in the interstitium of the alveolar wall, and a granulomatous inflammation enveloping airspace deposits of MWCNT agglomerates [9,33].

In our experiments we observed similar pulmonary inflammatory responses as reported in the literature following oropharyngeal aspiration of MWCNTs that peaked between 1 and 7 days and decreased by 28 days. This result is similar to that described in time of an acute inflammatory reaction which peaked at 7 days in the mouse and resolved over a 56 day period [7,9,34]. The pulmonary inflammatory response in our study may be related to how the MWCNTs were dispersed using a clinical grade animal-based lung surfactant. Previous reports have demonstrated that MWCNTs suspended in a natural lung surfactant or synthetic lung surfactants could influence both the lung dispersion and cytological effects of the CNTs [35-37]. In addition, the presence of proteins in the suspension that ultimately form a corona around the CNTs are believed to modulate MWCNT toxicity, phagocytosis and the inflammatory response [38,39].

Lung histopathology showed persistence of MWCNTs within the tissue that ultimately resulted in granuloma-like lesion formation at both low and high concentration at 28 days following instillation with each MWCNT type. Our finding was consistent with chronic granuloma formation reported earlier by us and others in rodent models [10,11].

Our study demonstrated a modest pulmonary inflammatory response but a more robust cardiac infarct exacerbation both in a time- and dose-dependent manner following oropharyngeal aspiration of MWCNTs. Infarct expansion remained evident at the highest concentration at 7 days post- oropharyngeal aspiration. Although we observed a diminution of the infarct size with all three forms of MWCNTs with time, the expanded infarct was still present as late as 28 days post- oropharyngeal aspiration. This type of result may underlie the importance of defining the endpoint of interest as to determining systemic toxicological response. Overall the results of our experiments contribute to the mounting evidence of the harmful effects of air pollution and engineered nanomaterials on the cardiovascular system [13,17,40]. Lack of blood supply or ischemia underlies many of the most important diseases encountered by physicians, including myocardial infarction, thrombotic stroke, embolic vascular occlusions, and peripheral vascular insufficiency. Evidence from animal studies suggests that reperfusion of ischemic areas may contribute to further tissue damage (I/R injury) [41]. I/R injury plays a key role in the pathophysiology of myocardial tissue damage after thrombolysis, angioplasty, or coronary artery bypass surgery [42-44]. We chose to investigate the effects of MWCNTs on an acute I/R injury model to simulate the occurrence of the common cardiovascular events in an acute setting. The relative uniformity in anatomy and physiological process found in the mouse model allows experimental interpretations that can be translated to the clinical setting [45]. As previously reported although the topographical left coronary artery in mice is not a functional left coronary artery as we understand it in humans, [45] and the mouse model as used to study the effects of ischemia reperfusion injury has been shown to be reproducible [45,46]. Our experiments used a 20-min occlusion period. Thirty minutes is a more common occlusion period in this model, but those studies of I/R injury are focused on mechanisms to reduce injury, and the occlusion time is selected to produce a sufficiently large infarction to permit measurable decreases associated with treatment. In our experiments, we hypothesized that treatment would exacerbate injury, and the occlusion time was shortened to enable an accurate reflection of expanded injury. The twenty-minute ligation of the LAD has been reported previously in the murine model [47,48].

Studies have investigated the dose- and time- dependent responses of pulmonary toxicity of MWCNTs after oropharyngeal aspiration in mice [9,49]. Our experiments illustrate a concentration- and time- dependent response of cardiac toxicity after oropharyngeal aspiration of MWCNTs. We observed a concentration-dependent effect with the N-doped MWCNTs but not with either C-grade or COOH MWCNTs. In contrast, the C-grade and COOH MWCNTs exhibited a threshold response with 1 μg MWCNT associated with a significant expansion of the infarction. Surprisingly, at one day following oropharyngeal aspiration of the N-doped MWCNT, the infarct was significantly expanded at of 0.1 μg.

The infarct exacerbation that we observed with the N-doped MWCNTs may reflect its unique physicochemical properties notably their bamboo-like structure and surface charge distribution resulting in the augmentation of the cardiovascular response. The presence of nitrogen atoms during the nanotube growth process is known to promote the growth of inner caps to yield structures that resemble bamboo sticks [50]. Previously, some studies found that N-doped MWCNTs exhibit different physiological response since the bamboo-like rough surfaces modify the van der Waal interactions between N-doped nanotubes [51,52]. N-atoms often dope the carbon lattice in non-graphitic configuration via the removal of an electron (pyridinc, pyrrolic). Hence, a high doping percentage of N (~19% in our case; see Table 1) may lead to the formation of non-graphitic (pyridinc/pyrrolic) phases that may act as localized charge centers. Previously, several researchers have demonstrated differences in cytotoxicity, hemo- and biocompatibility between pristine and N-doped MWCNTs [51,52]. However, the exact role of N atoms in the observation of such mitigated toxicity responses is yet to be understood. It is possible that the presence of large amount of N-atoms results in local charge centers. Such charge centers assist N-doped MWCNT interact more effectively with any polar entities compared to pristine or COOH functionalized forms. In fact, N-doped MWCNTs have been employed as electrodes in redox-active cells due to their unique charge distribution [53].

In addition to the potential role of nitrogen doping in contributing to a more robust cardiac response, surface area may also contribute to these differences. Physical characteristics of MWCNTs such as small size, large surface area and high reactivity, are cited as significant factors of their potential toxicity [12,27,32]. It has been postulated that the large surface area (250–300 mg/m^2^) of some MWCNTs may play a significant role in the development of pulmonary effects [54]. In our results, it is interesting to note that the most extensive cardiac injury and dose sensitivity was associated with the N-doped MWCNT that had the highest measured surface area. However, there was no significant difference in the surface area or hydrodynamic size estimates for the COOH and C-grade MWCNTs but there were differences in the time course of the cardiac I/R response. We believe that surface area may be another contributing factor along with the surface modification.

The exact mechanism by which MWCNTs may exhibit cardiovascular toxicity is unknown. Mechanisms of nanoparticle cardiovascular toxicity postulated in the literature include oxidative stress, inflammation, emanating in part, from pulmonary inflammation leading to atherothrombosis [55]; translocation of inhaled particles to the vasculature [56], direct effects on endothelial cells and cardiovascular system [57,58] and/or effects on the autonomic nervous system [55,59].

Systemic cytokine response to MWCNT oropharyngeal aspiration has not been adequately investigated although studies have reported cytokine analysis of BALF after pulmonary exposure to MWCNTs [11,36]. We therefore chose to study the circulating cytokine profile after oropharyngeal aspiration of MWCNTs. A close correlation between cytokines and I/R injury has been demonstrated [21]. IL-6, IL-10, IL-12, KC have been implicated in I/R injury after myocardial infarction [21,60,61]. IL-6 has been shown to contribute to cardiovascular dysfunction induced by acute lung injury [62]. MWCNTs and SWCNTs have been shown to provoke inflammation by induction of the pro-inflammatory genes IL-1β and IL-6 [63]. Although not statistically significant, we also found elevated levels of IL-6, IL-10, IL-12, IL-13, KC and MIP1-α with the COOH and the N-doped MWCNTs. IL-1β did not reveal any statistically significant differences compared to the SS and across the three treatment groups.

Wang and co-workers demonstrated that pulmonary exposure to MWCNTs induces an inflammatory response marked by increased levels of Eotaxin (Ccl11) that promote inflammatory and fibrotic changes observed within the lung [11]. We reported a statistically significant increase in the level of Eotaxin with the COOH exposed mice. Serum eotaxin levels have been associated with the etiologies of cardiac injury and heart failure as well as the regulation of inflammatory cell recruitment to infarcted myocardial tissue for wound repair [64-66]. The increase in the eosinophils with the COOH and the N-doped MWCNTs instillation in mice in the BALF observed in our experiments may suggest a possible link of Eotaxin to increased response of the myocardium to an imposed stress from I/R injury. The hematological assessment from these groups at 24 hours post oropharyngeal aspirations does not reflect a strong inflammatory either. As previously reported by Robertson there was no indication of systemic inflammation at 24 hours after diesel exhaust particle instillation although there were increased levels of inflammatory mediators at 6 hours [67]. While there was significant elevation in a common marker of inflammation in the number of neutrophils from the COOH exposed mice but overall WBC numbers remained in the normal range and suggest there is no significant inflammatory state. Any conclusion drawn from these observations must be viewed with caution as the cytokines and blood cells counts are known to rapidly change following an acute pulmonary exposures and we do not provide direct evidence for the role of these cytokines exacerbating the I/R injury. It still remains a hypothesis that circulating cytokines may prime target organs to response more robustly to any imposed injury.

As previously demonstrated by Cozzi et al, oropharyngeal aspiration of mice with particulate matter increased cardiac oxidative stress within the myocardium after I/R injury [68]. Furthermore, it has also been shown that carbon nanotubes in general can lead to reactive oxygen species (ROS) generation [69]. Xia and co-workers have shown that redox cycling can contribute to ROS production. This can occur because of the presence of transition metals or redox cycling organic chemicals on the particle surface [70]. Moreover, transition metals can generate hydroxyl radicals through the Fenton reaction [71]. The Fenton reaction is one of the mechanisms by which metal impurities on or in the CNT may induce ROS production [70]. Although we do not have any evidence, it is plausible that ROS production could contribute to some of the infarct exacerbation that we observed in our experiments.

Translocation and extrapulmonary toxicities of MWCNTs were demonstrated by Reddy et al [72] while Aiso and co-workers have shown translocation of intratracheally instilled MWCNTs to lung associated lymph nodes [73] such evidence may suggest a close association with the circulatory system following exposure. Although there is a lack of definitive studies on the translocation of MWCNTs leading to direct particle effect on the cardiac myocytes after oropharyngeal instillation, it could be hypothesized that MWCNTs could promote infarct exacerbation based on the underlying translocation mechanisms as demonstrated in the above mentioned studies.

In summary, our results show that oropharyngeal aspiration of MWCNTs in mice exacerbates myocardial infarction with an increase in infarct size in a concentration-, time- and surface modification-dependent manner. This study was the first to assess the impact of adverse outcomes of exposure to MWCNTs on the susceptibility of cardiac tissue to ischemic injury. However, the mechanisms by which MWCNTs exposure generates cardiac tissue that is susceptible to injury secondary to MWCNTs oropharyngeal aspiration remains to be determined. The extent of I/R injury seen at low doses, where there is a lack of robust pulmonary response, is significant and suggests that the cardiovascular impact should be considered when assessing the safety of engineered nanomaterials. Taken together, the findings are of sufficient significance to warrant continued studies to evaluate the systemic effects of MWCNTs associated with various exposure conditions.

## Materials and Methods

### Carbon Nanotube Characterization

Three forms of chemical, vapor-deposition grown multi-walled carbon nanotubes (MWCNTs) were generously provided by NanoTech Labs (Yadkinville, NC). The three forms included an unmodified commercial grade (C-grade), acid treated carboxylated (COOH) and nitrogen doped (N-doped) forms. The MWCNTs were suspended in a 10% Infrasurf™ surfactant/sterile normal saline solution (a generous gift of ONY Inc. Amherst, NY) and subjected to bath-sonication for 45 minutes (Branson Sonicator, model 2510, Branson Ultrasonics Corp, Danbury, CT) prior to characterization.

### Electron microscopy

Detailed scanning and transmission electron microscopy were performed using Hitachi S-4800 and 9500 microscopes to define the length and diameter distribution of the MWCNTs.

### Surface area measurements

The surface areas and pore volumes of the dehydrated MWCNT suspensions were obtained using a physisorption analyzer (Micromeritics ASAP 2010) based on the Brunauer-Emmett-Teller (BET) equation [19] and the Barrett–Joyner–Halenda (BJH) method [20].

### Thermogravimetric analysis (TGA)

Following the EDX (Energy Dispersion Xray) detection of Fe presence, TGA analysis was performed on each of MWCNTs to quantify the amount of residual Fe present. The measurements were carried out using a Perkin Elmer Thermogravimetric Analyzer (Pyris 1 TGA). Approximately 40 - 50% of the volume of the platinum pan was filled with MWCNT (0.1 - 0.2 cm^3^). The sample was heated from 30°C - 800°C at a rate of 20°C/minute in an air atmosphere using an air flow rate of 20 ml/minute.

### Raman Spectroscopy

Raman spectra were collected using a Dilor XY triple grating spectrometer equipped with a TE cooled CCD at 514.5 nm Ar^+^ ion excitation Raman spectroscopy has been used widely to determine the MWCNT quality using the disorder-induced (D-band) and graphitic (G-band) bands. The ratio (R) of D-band to the G-band integrated area (*R=I*_*D*_*/I*_*G*_) is often used to quantify the defects in carbon nanostructures.

### Dynamic Light Scattering

Hydrodynamic size distributions of the MWCNT suspensions were measured using a dynamic light scattering device (Malvern Instruments, Nanosizer S90). The surface charges (and therefore suspension stability) and isoeletric points (IEP) of the MWCNT suspensions were also determined using a zeta potential device (Malvern Instruments, Zeta ZS).

### Animals

6 - 8 week old male C57BL/6J mice were obtained from Jackson Labs (Bar Harbor, Maine, USA) and were housed and cared for by the East Carolina University Department of Comparative Medicine Staff. All protocols conformed to the standards in the National Institute of Health’s Guide for the Care and Use of Laboratory Animals and were approved by the East Carolina University Institutional Animal Care and Use Committee. Animals were housed on a 12/12 h light dark light cycle and provide water and food *ad libitum* prior to experimental manipulation and allowed to acclimate for 5 days prior to exposure.

### MWCNT exposure

Each of the three types of MWCNTs (C-grade, COOH and N-doped) were suspended in 10% Surfactant-Saline (SS) as concentrations of 0.01, 0.1, 1, 10 and 100 μg MWCNT per 100 μl suspension and orpharyngeal aspiration was performed under light Isoflurane anesthesia. Prior to oropharyngeal aspiration in mice, the MWCNTs were bath sonicated using a cuphorn sonicator (Misonix Model S-4000 Qsonica LLC Newton, CT) for 4 mins at an amplitude of 65 and total of 20,807 Joules of energy. Animals underwent I/R injury protocol at 1, 7 and 28 days following exposure for pulmonary and cardiovascular studies.

### BAL collection and lung histology

Bronchoalveolar lavage was performed on the right lung at 1 day, 7 days and 28 days post-exposure for the 1 μg and 100 μg of the three forms of MWCNTs. The right lung was lavaged *in situ* four times with ice-cold Hanks balanced salt solution (26.25 ml/ kg body weight). The first aliquot of bronchoalveolar lavage fluid (BALF) was collected separately for cytokine analysis, while aliquots 2 - 4 were pooled for cell count and protein. All BALF was centrifuged at 1000 x g for 10 minutes at 4°C. Total cells from pooled lavage aliquots were collected and 20,000 cells were centrifuged using a Cytospin IV (Shandon Scientific Ltd., Cheshire, UK) and stained with a three-step hematology stain (Richard Allan Scientific, Kalamazoo, MI, USA). Cell differential counts were determined by assessing morphology microscopically with evaluation of 300 cells per slide [11].

### Lung Histopathology

To determine histopathologic alterations, unlavaged left lungs were collected from mice exposed to 1 μg or 100 μg each of the C-grade, COOH and N-doped MWCNTs or from SS instilled mice. Lungs were gently inflated and perfused with 10% neutral buffered formalin fixative and stored for 24 - 72 hours at 4°C. Lungs were embedded in paraffin and 8 μM cross-sections were mounted on slides. Sections were stained with hematoxylin and eosin (H&E) to identify tissue histological changes and MWCNT deposition [11].

### Hematology and WBC Counts

Total and differential leukocyte count, red blood cell count and platelet count were performed on mice after oropharyngeal aspiration of 100 μg of each of the three forms of MWCNTs, C-grade, COOH and N-doped. The cell counts were performed on animals not subjected to I/R protocol. The blood counts were performed on 100 μl of whole blood using a Coulter Counter Analyzer Model S (Coulter Corporation, Miami, Fl ) and stained with Quick Slide Stain Pack (GG&B Co., Wichita Falls, TX). Two slides were prepared for each animal, 100 cells were counted per slide, counts from each animal were averaged and the average for all animals was reported.

### Serum Cytokine Analysis

Serum cytokine profile analysis was performed from blood samples collected 1 day post- oropharyngeal aspiration of 100 μg MWCNTs (C-grade, COOH or N-doped) and SS. The Milliplex multi-analytes kit included pro-inflammatory cytokines and chemotactic factors, (Lincoplex Cytokine/Chemokine 20 plex kit Cat #: MPXMCYTO70KPMX220. The following analytes were detected and measurable: Eotaxin, IL-5, IL-6, IL-10, IL-12 (p40), IL-12(p70), IL-13, KC, MCP-1, MIP-1α and β, MIP-2 and RANTES. A serum sample was also analyzed form a separate group of animals for cytokines IL-1β and TNF-α using the ELISA assay (R&D systems Inc, Minneapolis, MN). Blood samples were collected 1 day oropharyngeal aspiration of 100 μg MWCNTs (C-grade, COOH or N-doped) and SS. The results for IL-1β and TNF-α are included in Table 6.

### Ischemia Reperfusion (I/R) Injury Protocol

I/R injury was accomplished using an adaptation of the protocol and as reported previously by Cozzi and Hazarika et al [47,68]. After oropharyngeal aspiration with MWCNT or SS, mice were anesthetized by an intraperitoneal (*i.p.)* injection of Ketamine/Xylazine (18:2 mg/ml ratio with 0.05 ml/10g body weight). A midline tracheostomy was performed, and the animals were intubated with #22 angiocath tubing and mechanically ventilated with 100% oxygen using a Harvard Inspira ASV ventilator (Harvard Apparatus, Holliston, MA). The initial ventilator settings were set according to body weight tables, and adequacy of ventilation was determined by direct visual confirmation of satisfactory lung expansion through the subsequent open thoracotomy. Supplemental injection of the anesthetic mixture was provided as needed during the surgical procedure to maintain anesthesia. Body temperature was maintained at 37°C with a TC-1000 temperature controller and heating pad (CWE, Inc, Ardmore, PA).

A left parasternal thoracotomy was performed and the pericardium was gently removed from the heart. The left anterior descending coronary artery (LAD) was identified and ligated with #6-0 prolene over a #90 PE tubing ~ 4 mm distal to the origin between the conus arteriosus and the left atrium. The location of the ligature was chosen based on preliminary experiments indicating that occlusion at this level placed ~ 50% of the left ventricle at risk of ischemic injury. Effective occlusion was confirmed visually by both the presence of pallor and dyskinesia distal to the ligature. After 20 min of ischemia the PE tubing was removed and reperfusion was allowed for 2 h. The ischemic time was chosen based on preliminary studies where 20 min of occlusion resulted in a reproducible infarct size in control animals that allowed for potential infarct expansion without increased mortality [47].

After the reperfusion period, the LAD was re-ligated at the original point of occlusion and 1% Evan’s blue dye was delivered via aortic arch cannulation to delineate the perfused myocardium form the non-perfused myocardium during ischemia [68]. After Evan’s blue staining, the hearts were excised and 1 mm thick serial sections were cut from the point of ligation to the apex.

### Determination of Infarct size

Heart sections were incubated for a minimum of 20 min in a 0.1% solution of 2, 3, 5-triphenyltetrazolium chloride (TTC) to identify the infarcted from the non-infarcted myocardium [68]. Both sides of all the sections were digitally imaged and analyzed using computer planimetry software (NIH Image J) to quantify left ventricular (LV) area, area at risk (AAR) and infarcted area. The extent of infarct was expressed as a percent of AAR [68].

### Statistical Analysis

Data are reported as mean ± standard error of mean (SEM). Differences in dose-response and time points between the three groups of MWCNT were analyzed using Microsoft Excel and Prism 5 (Graph Pad Software Inc, La Jolla, CA). Statistical significance was determined by one-way ANOVA with Tukey’s multiple comparison test. A *P* value ≤ 0.05 was considered statistically significant.

## Competing interests

The authors declare that they have no competing interests.

## Authors’ contributions

RNU carried out the I/R experimental design and drafted the manuscript. EM, PK, XW and SH preformed the BAL and cytokine assays and assisted in data analysis and interpretation. RP and AMR performed the SEM, TEM, Raman spectroscopy and TGA analysis of the MWCNTs. BSH participated in initial experiment design and MWCNT characterization. PC and PCK determined the MWCNT surface area, pore size, IEP and zeta potential. RML, JMB and CJW participated in the study design, data interpretation and editing of the manuscript. CJW conceived the study design and was responsible for the coordination of the experiments and writing of the manuscript. All authors read and approved of the final manuscript.

## Supplementary Material

Additional file 1 **Figure S1: Representative Raman spectra for the 3 MWCNT forms.** Red traces reports raw signal and black lines fitted curve to the signal. The Raman spectrum of MWCNT obtained using 514.5 nm laser excitation. The presence of strong disorder band (I_D_/I_G_ peak ratio) suggests the existence of structural defects as determined by Raman spectroscopy. Traces show the presence of the D and G band peaks and the ratios of the strengths of these bands (R = I_D_/I_G_) are used to quantify defects in the carbon nanostructure and reflect the defects in the functionalized COOH and N-doped versions of the MWCNT. **Figure S2**: Representative SEM image of C-grade MWCNT bundle with an individual fiber length 38 μm. (adapted from adapted form Wang et al, Part Fibre Toxicol. 2011:8:24). A detailed scanning electron microscopic study reveals the lengths of indvidual C-grade MWCNTs to be in the range of 10-100 μm. **Figure S3**: The diameter distribution of C-grade MWCNTs (adapted form Wang et al, Part Fibre Toxicol. 2011:8:24 ) Fiber diameter distribution based, on TEM measurements, found a a bi-modal distribution with peak diameters at ~12.5 nm and 27.5 nm. **Figure S4:** Representative report generated for the C-grade form of the MWCNT including SEM image, spectrum region, EDX elemental spectra and Table with calculated percent weight and percent atoms of the elemental components. **Figure S5**: A Representative SEM image of COOH MWCNT bundle with a individual length ~78 μm. A detailed scanning electron microscopic study reveals the lengths of indvidual COOH MWCNTs to be in the range of 40-100 μm. **Figure S6**: The diameter distribution for COOH MWCNTs. Carboxylated MWCNTs were found have a bi-modal diameter distribution similar to C-grade MWCNTs with peaks ~15 nm and ~30 nm. Such similar results are expected since the carboxylation process (performed on C-grade MWCNTs) does not change the diameter distribution significantly. Fiber diameter distribution based, on TEM measurements. **Figure S7:** Representative report generated for the COOH form of the MWCNT including SEM image, spectrum region, EDX elemental spectra and Table with calculated percent weight and percent atoms of the elemental components. **Figure S8**: A representative SEM image of N-doped MWCNTs bundle with an individual fiber length ~84 μm. A detailed scanning electron microscopic study reveals the lengths of indvidual N-doped MWCNTs to be in the range of 50-80 μm. **Figure S9**: The diameter distribution for N Doped MWCNTs Fiber diameter distribution based, on TEM measurements. In the case of N-doped TEM studies indicate a unimodal diameter distribution with peak at ~22.5 nm. **Figure S10**: Representative report generated for the N-doped form of the MWCNT including SEM image, spectrum region, EDX elemental spectra and Table with calculated percent weight and percent atoms of the elemental components. **Figure S11**: Histopathology of lungs 1 day post oropharyngeal aspiration of MWCNT displays modest inflammatory response and distributed deposition of MWCNT. Mice exposed to 10% surfactant vehicle in saline (SS) control display normal lung morphology while mice exposed to 1 or 100 μg MWCNTs exhibit widely dispersed deposition of MWCNT aggregates within lung tissue. Agglomerates of MWCNT are indicated by arrows. H&E staining demonstrates peribronchioloar inflammatory foci in lungs of mice exposed to MWCNT but not vehicle. Images are representative of 4 mice per group with larger panels magnifications of 200x and insets at 400x. **Figure S12**: Histopathology of lungs 7 day post oropharyngeal aspiration of MWCNT displays modest inflammatory response and distributed deposition of MWCNT. Mice exposed to 10% surfactant in saline (SS) control display normal lung morphology while mice exposed to 1 or 100 μg MWCNTs exhibit continued presence of widely dispersed deposition of MWCNT aggregates within lung tissue. Agglomerates of MWCNT are indicated by arrows. H&E staining demonstrates peribronchioloar and alveolar wall inflammatory foci in lungs of mice exposed to MWCNT but not vehicle. Images are representative of 4 mice per group with larger panels magnifications of 200x and insets at 400x. **Figure 13**: Histopathology of lungs 28 day post oropharyngeal aspiration of MWCNT displays modest inflammatory response with **granulomatous and fibrotic tissue response** and persistent distributed deposition of MWCNT. Mice exposed to 10% surfactant in saline (SS) control display normal lung morphology while mice exposed to 1 or 100 μg MWCNTs exhibit continued presence of widely dispersed deposition of MWCNT aggregates within lung tissue. Agglomerates of MWCNT are indicated by arrows. H&E staining demonstrates peribronchioloar and alveolar wall inflammatory foci in lungs of mice exposed to MWCNT but not vehicle. Images are representative of 4 mice per group with larger panels magnifications of 200x and insets at 400x. Click here for file
