# Supplementary material for: Expansion of cardiac ischemia/reperfusion injury after instillation of three forms of multi-walled carbon nanotubes
Source: Part Fibre Toxicol. 2012 Oct 16;9:38. doi: 10.1186/1743-8977-9-38 (PMC3518151; doi:10.1186/1743-8977-9-38)
Supplement: Additional file 1 — Figure S1: Representative Raman spectra for the 3 MWCNT forms. Red traces reports raw signal and black lines fitted curve to the signal. The Raman spectrum of MWCNT obtained using 514.5 nm laser excitation. The presence of strong disorder band (ID/IG peak ratio) suggests the existence of structural defects as determined by Raman spectroscopy. Traces show the presence of the D and G band peaks and the ratios of the strengths of these bands (R = ID/IG) are used to quantify defects in the carbon nanostructure and reflect the defects in the functionalized COOH and N-doped versions of the MWCNT. Figure S2: Representative SEM image of C-grade MWCNT bundle with an individual fiber length 38 μm. (adapted from adapted form Wang et al, Part Fibre Toxicol. 2011:8:24). A detailed scanning electron microscopic study reveals the lengths of indvidual C-grade MWCNTs to be in the range of 10-100 μm. Figure S3: The diameter distribution of C-grade MWCNTs (adapted form Wang et al, Part Fibre Toxicol. 2011:8:24 ) Fiber diameter distribution based, on TEM measurements, found a a bi-modal distribution with peak diameters at ~12.5 nm and 27.5 nm. Figure S4: Representative report generated for the C-grade form of the MWCNT including SEM image, spectrum region, EDX elemental spectra and Table with calculated percent weight and percent atoms of the elemental components. Figure S5: A Representative SEM image of COOH MWCNT bundle with a individual length ~78 μm. A detailed scanning electron microscopic study reveals the lengths of indvidual COOH MWCNTs to be in the range of 40-100 μm. Figure S6: The diameter distribution for COOH MWCNTs. Carboxylated MWCNTs were found have a bi-modal diameter distribution similar to C-grade MWCNTs with peaks ~15 nm and ~30 nm. Such similar results are expected since the carboxylation process (performed on C-grade MWCNTs) does not change the diameter distribution significantly. Fiber diameter distribution based, on TEM measurements. Figure S7: Representative re [file 1743-8977-9-38-S1.docx]

**Appendix 1**

**Raman Spectroscopy Results:**


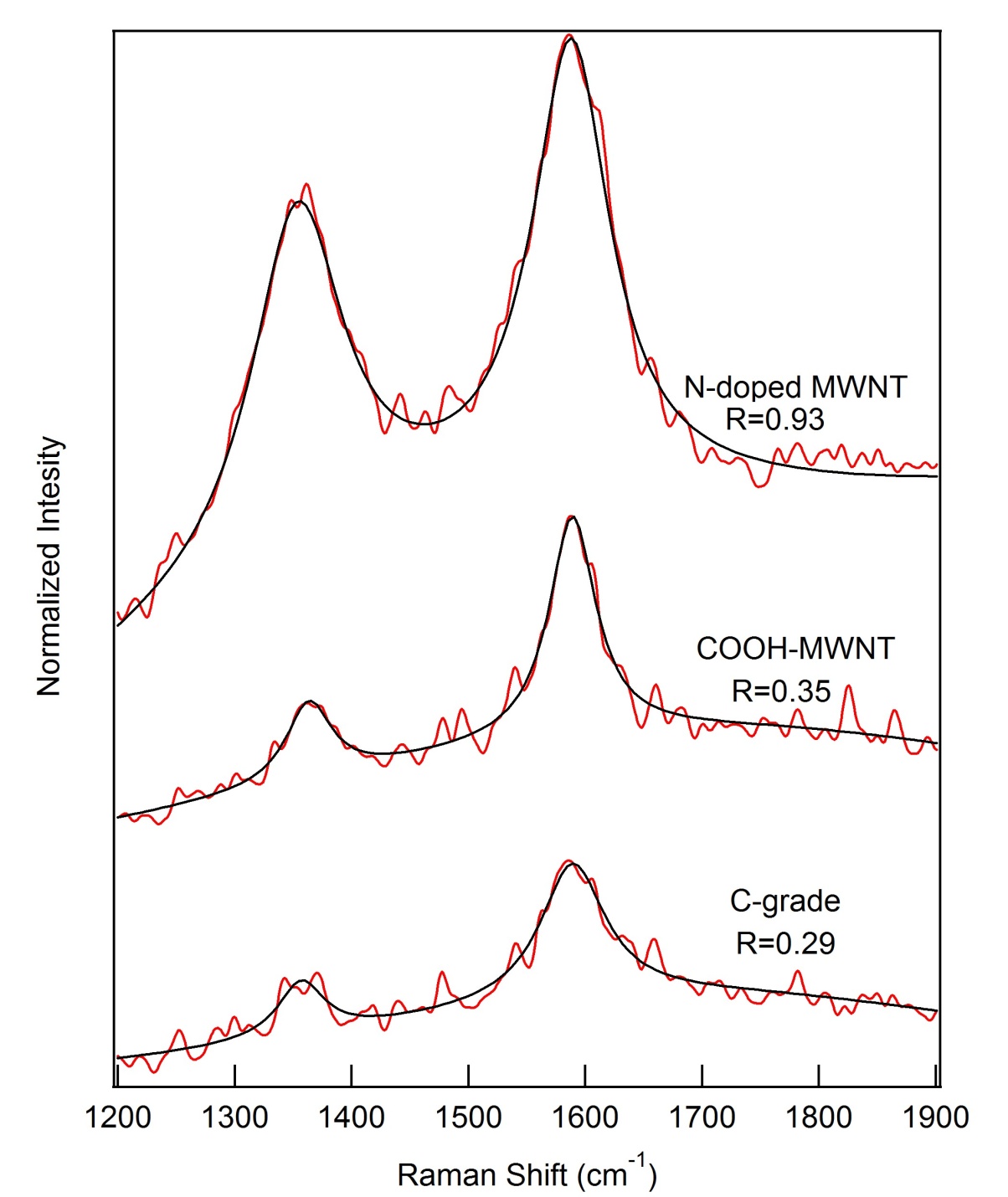


**Supplemental Figure 1**: Representative Raman spectra for the 3 MWCNT forms. Red traces reports raw signal and black lines fitted curve to the signal. The Raman spectrum of MWCNT obtained using 514.5 nm laser excitation. The presence of strong disorder band (I_D_/I_G_ peak ratio) suggests the existence of structural defects as determined by Raman spectroscopy. Traces show the presence of the D and G band peaks and the ratios of the strengths of these bands (R = I_D_/I_G_) are used to quantify defects in the carbon nanostructure and reflect the defects in the functionalized COOH and N-doped versions of the MWCNT.

**Electron Microscopy Studies:**

Commercial grade (C-grade) MWCNT:


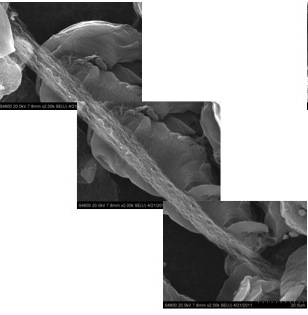


**Supplemental Figure 2**: Representative SEM image of C-grade MWCNT bundle with an individual fiber length 38 μm. (adapted from adapted form Wang et al, Part Fibre Toxicol. 2011:8:24). A detailed scanning electron microscopic study reveals the lengths of indvidual C-grade MWCNTs to be in the range of 10-100 μm.

C-grade Fiber Diameter Distribution:


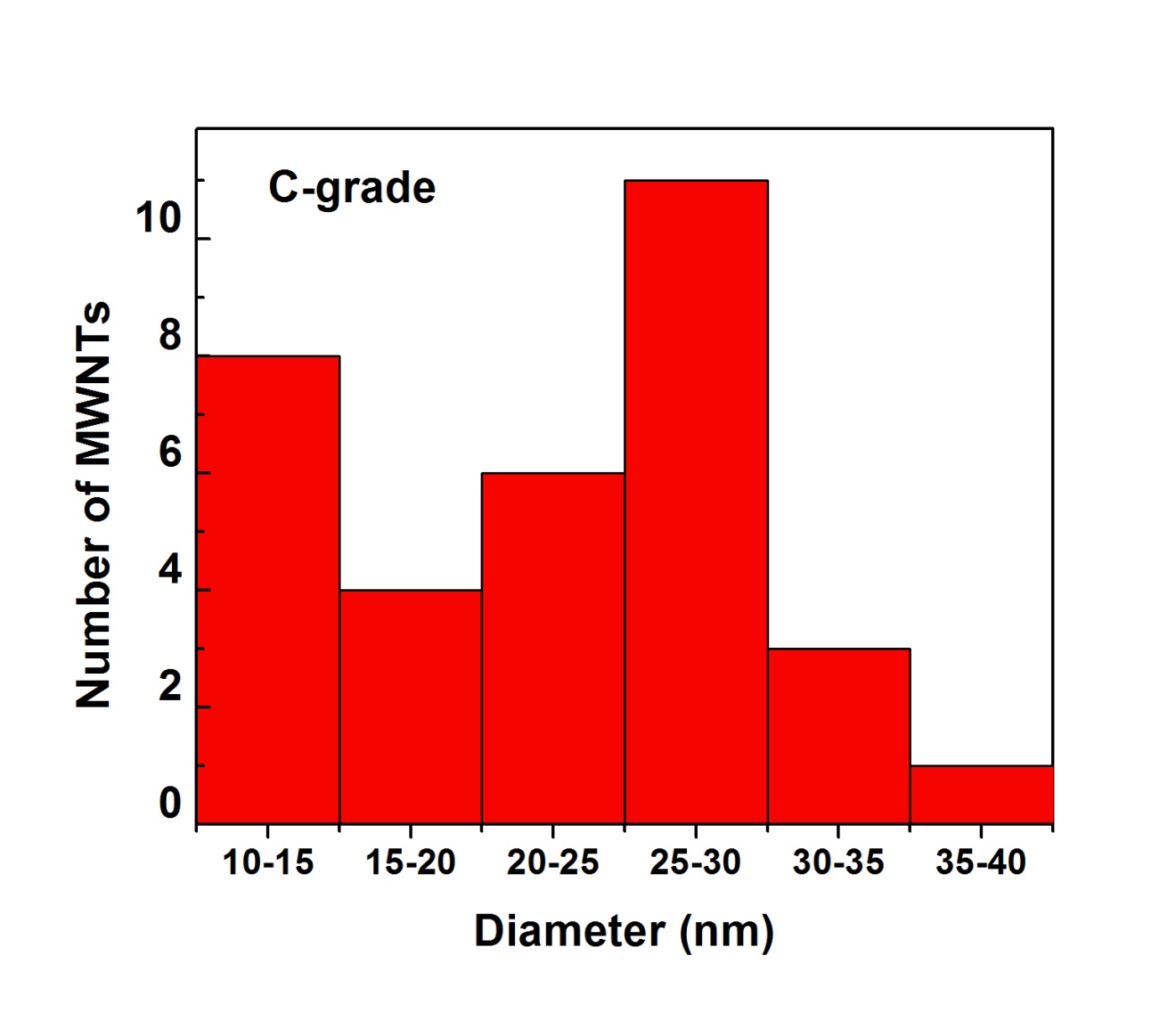


**Supplemental Figure 3**: The diameter distribution of C-grade MWCNTs (adapted form Wang et al, Part Fibre Toxicol. 2011:8:24 ) Fiber diameter distribution based, on TEM measurements, found a a bi-modal distribution with peak diameters at ~12.5 nm and 27.5 nm.

Representative Elemental Anaylsis of C-grade MWCNT:

**Supplemental Figure 4:** Representative report generated for the C-grade form of the MWCNT including SEM image, spectrum region, EDX elemental spectra and Table with calculated percent weight and percent atoms of the elemental components.

Carboxylated (COOH) MWCNTs:


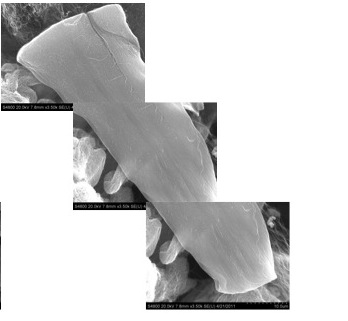


**Supplemental Figure 5**: A Representative SEM image of COOH MWCNT bundle with a individual length ~78 μm. A detailed scanning electron microscopic study reveals the lengths of indvidual COOH MWCNTs to be in the range of 40-100 μm.

COOH Diameter Distribution:


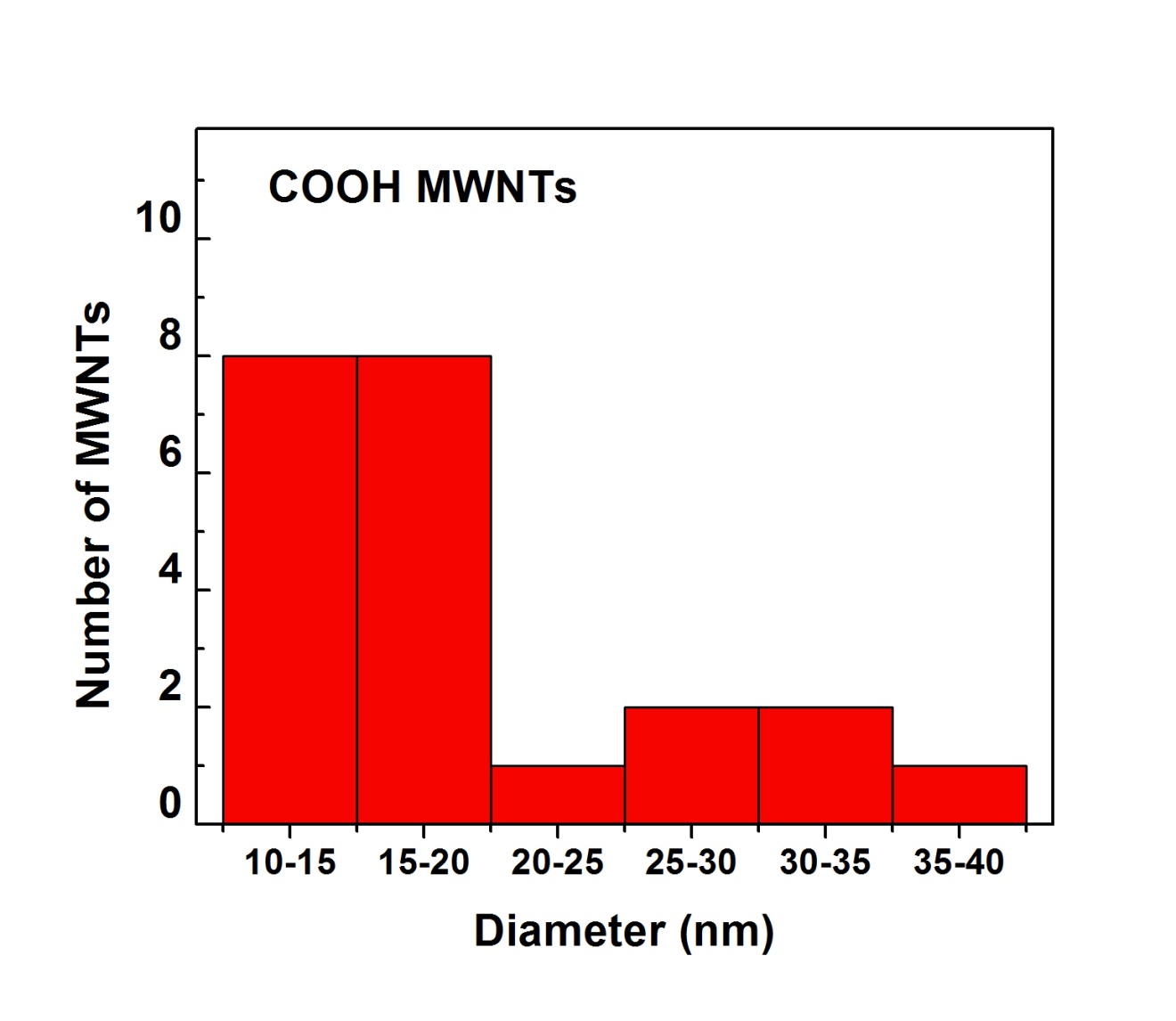


**Supplemental Figure 6**: The diameter distribution for COOH MWCNTs. Carboxylated MWCNTs were found have a bi-modal diameter distribution similar to C-grade MWCNTs with peaks ~15 nm and ~30 nm. Such similar results are expected since the carboxylation process (performed on C-grade MWCNTs) does not change the diameter distribution significantly. Fiber diameter distribution based, on TEM measurements.

Representative Elemental Anaylsis of COOH MWCNT:

**Supplemental Figure 7:** Representative report generated for the COOH form of the MWCNT including SEM image, spectrum region, EDX elemental spectra and Table with calculated percent weight and percent atoms of the elemental components.

N-Doped MWCNTs:


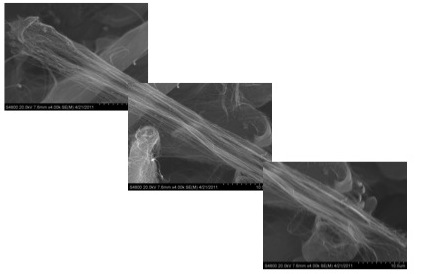


**Supplemental Figure 8**: A representative SEM image of N-doped MWCNTs bundle with an individual fiber length ~84 μm. A detailed scanning electron microscopic study reveals the lengths of indvidual N-doped MWCNTs to be in the range of 50-80 μm.

N doped MWCNT Diameter Distribution:


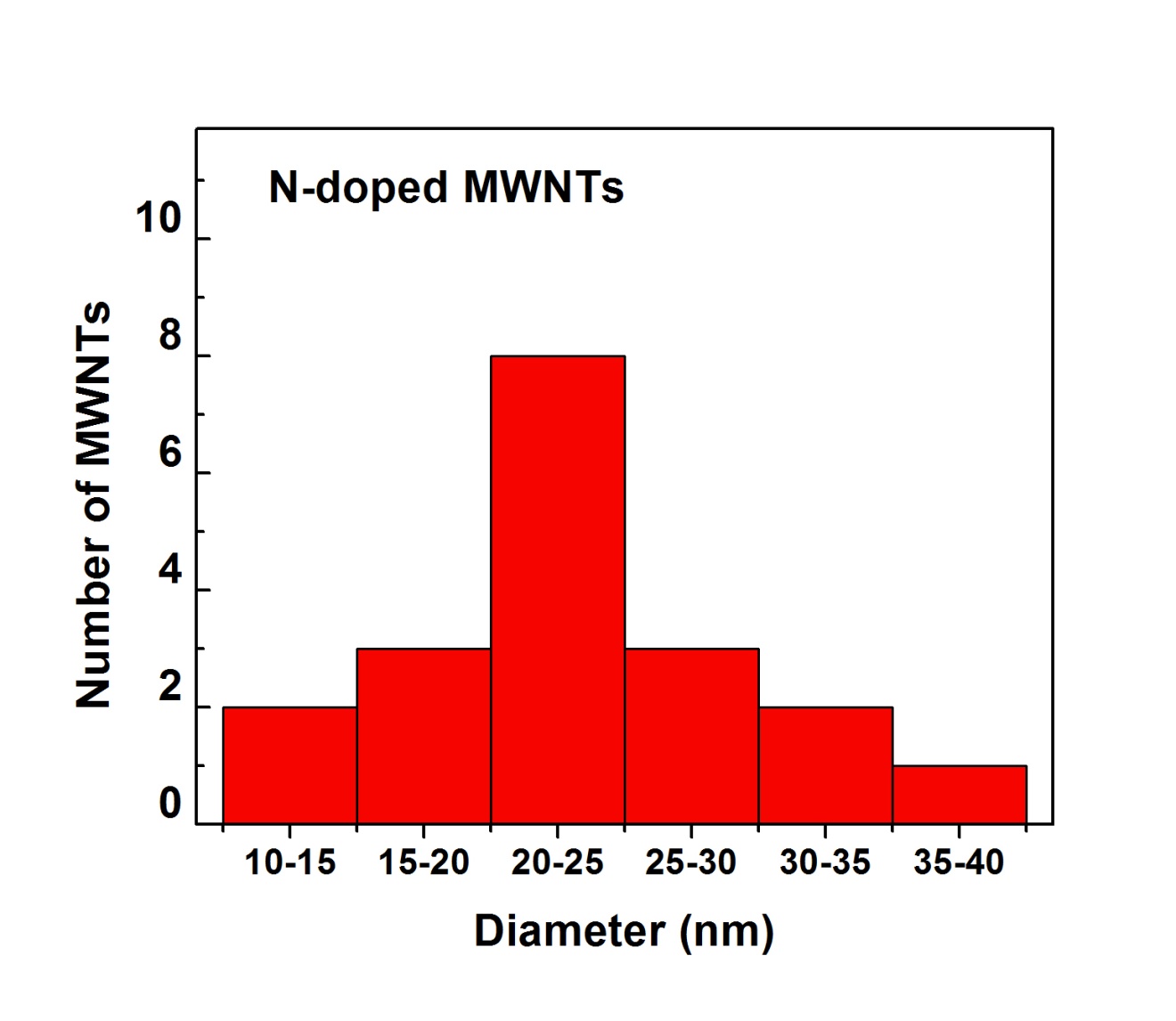


**Supplemental Figure 9**: The diameter distribution for N Doped MWCNTs Fiber diameter distribution based, on TEM measurements. In the case of N-doped TEM studies indicate a unimodal diameter distribution with peak at ~22.5 nm.

Representative Elemental Anaylsis of N doped MWCNT:

**Supplemental Figure 10**: Representative report generated for the N-doped form of the MWCNT including SEM image, spectrum region, EDX elemental spectra and Table with calculated percent weight and percent atoms of the elemental components.


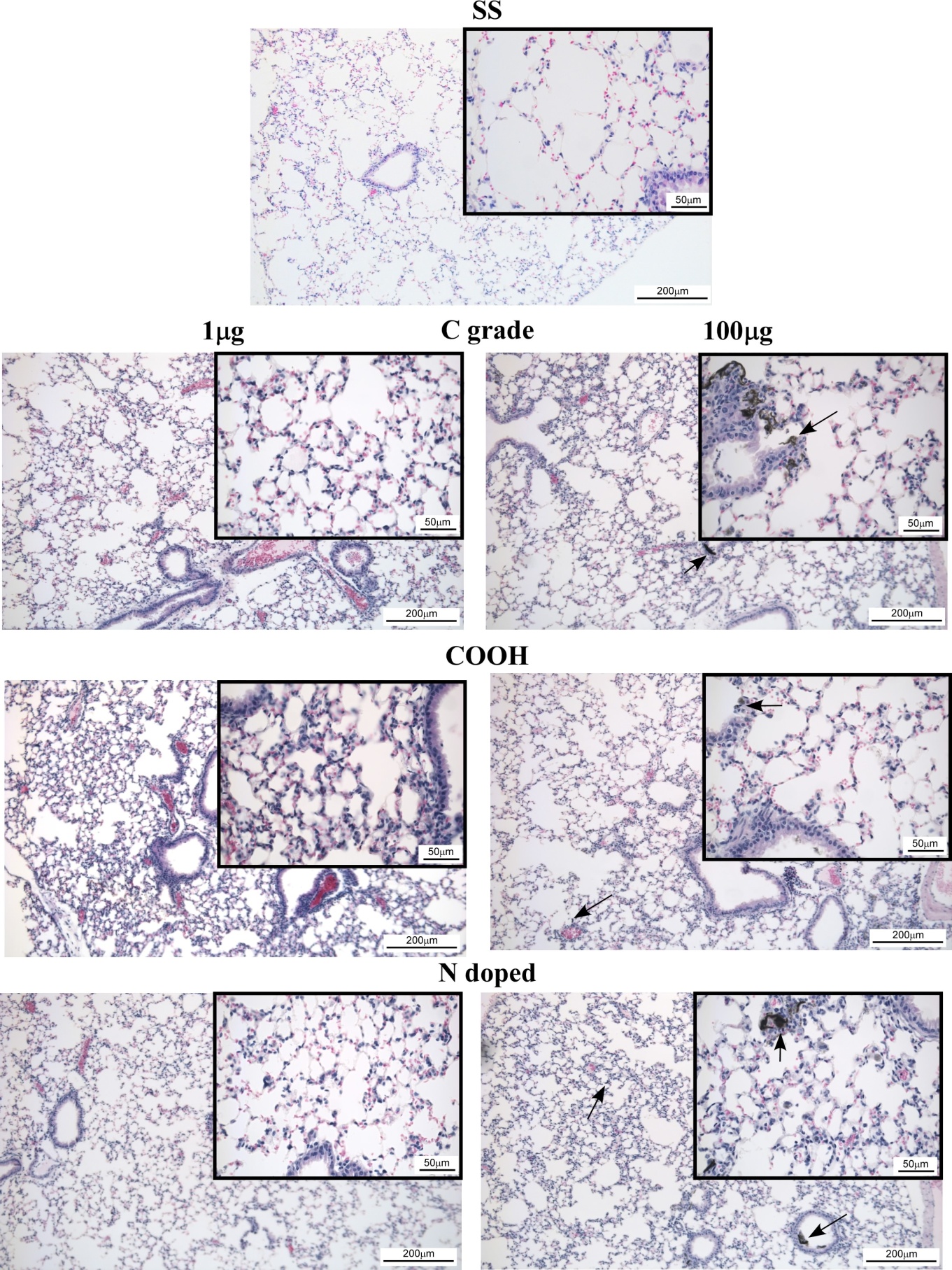


**Supplimental Figure 11**: Histopathology of lungs 1 day post oropharyngeal aspiration of MWCNT displays modest inflammatory response and distributed deposition of MWCNT. Mice instilled with 10% surfactant vehicle in saline (SS) control display normal lung morphology while mice instilled with 1 or 100 μg MWCNTs exhibit widely dispersed deposition of MWCNT aggregates within lung tissue. Agglomerates of MWCNT are indicated by arrows. H&E staining demonstrates peribronchioloar inflammatory foci in lungs of mice exposed to MWCNT but not vehicle. Images are representative of 4 mice per group with larger panels magnifications of 200x and insets at 400x.


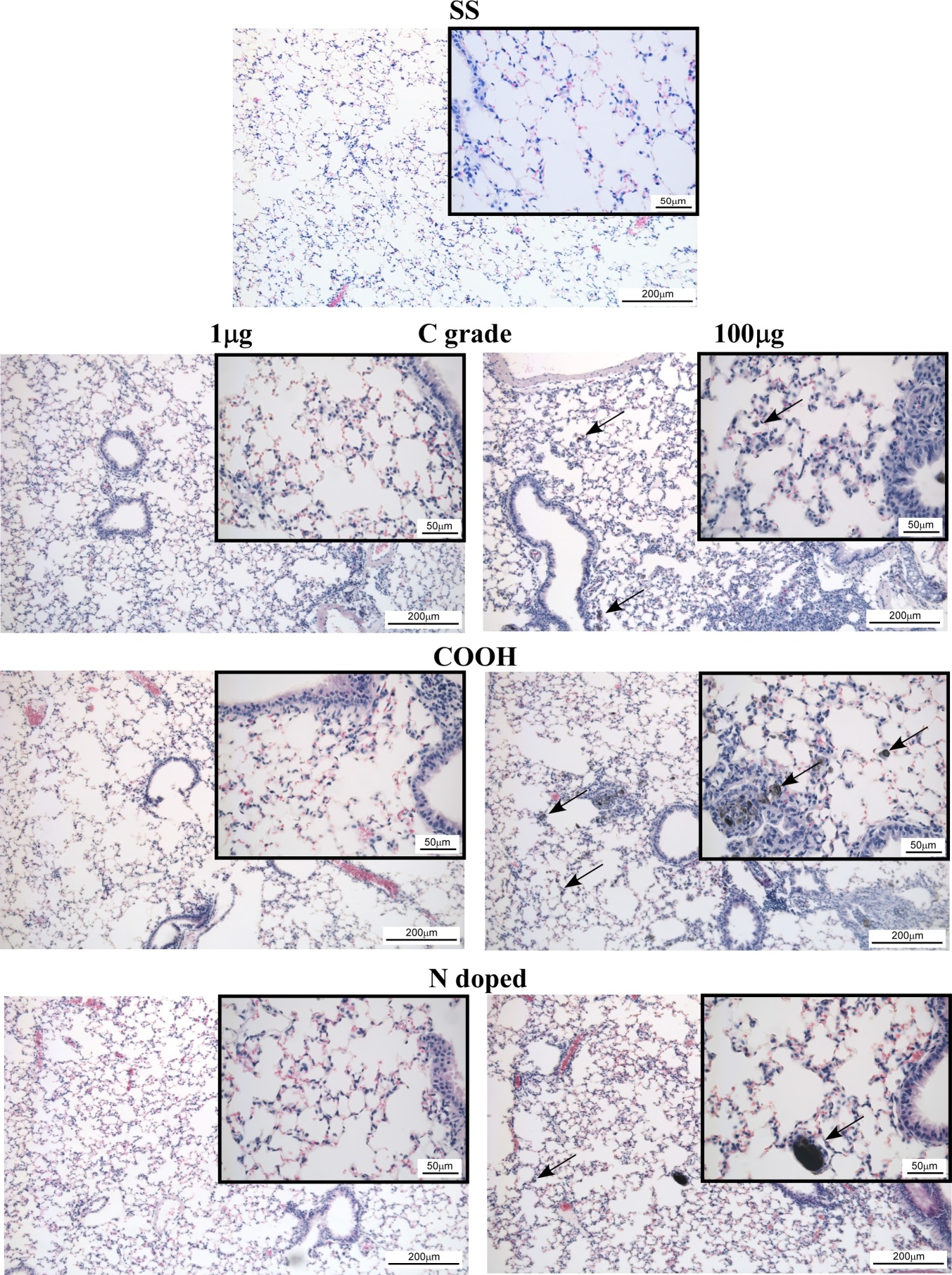


**Supplemental Figure 12**: Histopathology of lungs 7 day post oropharyngeal aspiration of MWCNT displays modest inflammatory response and distributed deposition of MWCNT. Mice instilled with 10% surfactant in saline (SS) control display normal lung morphology while mice instilled with 1 or 100 μg MWCNTs exhibit continued presence of widely dispersed deposition of MWCNT aggregates within lung tissue. Agglomerates of MWCNT are indicated by arrows. H&E staining demonstrates peribronchioloar and alveolar wall inflammatory foci in lungs of mice exposed to MWCNT but not vehicle. Images are representative of 4 mice per group with larger panels magnifications of 200x and insets at 400x.


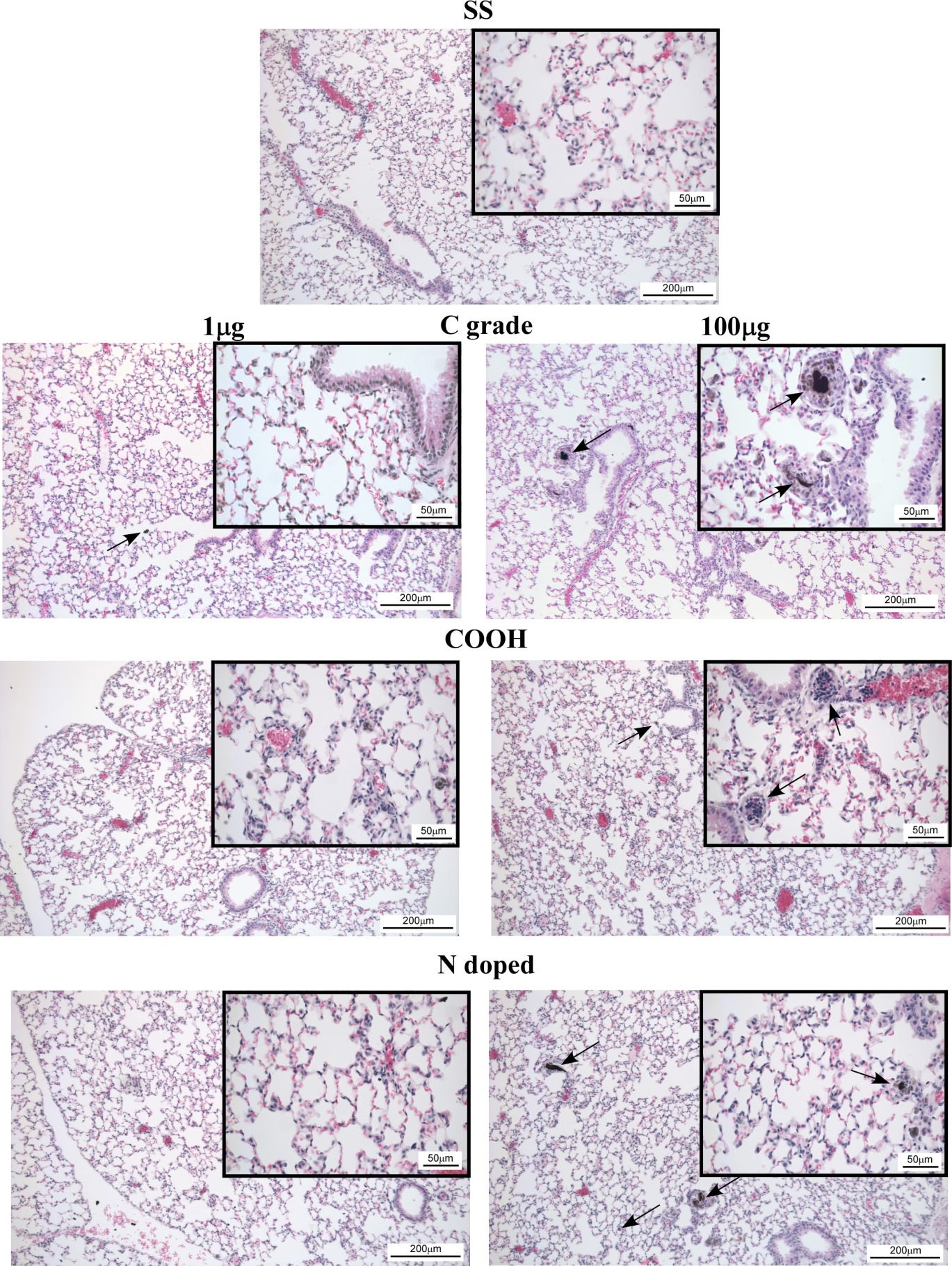


**Supplemental Figure 13**: Histopathology of lungs 28 day post oropharyngeal aspiration of MWCNT displays modest inflammatory response with **granulomatous and fibrotic tissue response** and persistent distributed deposition of MWCNT. Mice instilled with 10% surfactant in saline (SS) control display normal lung morphology while mice instilled with 1 or 100 μg MWCNTs exhibit continued presence of widely dispersed deposition of MWCNT aggregates within lung tissue. Agglomerates of MWCNT are indicated by arrows. H&E staining demonstrates peribronchioloar and alveolar wall inflammatory foci in lungs of mice exposed to MWCNT but not vehicle. Images are representative of 4 mice per group with larger panels magnifications of 200x and insets at 400x.
